# An Empirical Analysis of Rural-Urban Differences in Out-Of-Pocket Health Expenditures in a Low-Income Society of China

**DOI:** 10.1371/journal.pone.0154563

**Published:** 2016-05-25

**Authors:** Lidan Wang, Anjue Wang, Detong Zhou, Gerry FitzGerald, Dongqing Ye, Qicheng Jiang

**Affiliations:** 1 School of Health Management, Anhui Medical University, No.81, Mei Shan Road, Hefei 230032, Anhui, China; 2 School of Public Health, Anhui Medical University, No.81, Mei Shan Road, Hefei, 230032, Anhui, China; 3 Department of Development and Planning, Anhui Medical University, No.81, Mei Shan Road, Hefei, 230032, Anhui, China; 4 Survey Office of the National Bureau of Statistics in Anhui, No.168, Wu Hu Road, Hefei, 230001, Anhui, China; 5 School of Public Health and Social Work, Queensland University of Technology, Victoria Park Road, Kelvin Grove, Brisbane, 4059, Australia; Tulane University School of Public Health, UNITED STATES

## Abstract

**Objective:**

The paper examines whether out-of-pocket health care expenditure also has regional discrepancies, comparing to the equity between urban and rural areas, and across households.

**Method:**

Sampled data were derived from Urban Household Survey and Rural Household Survey data for 2011/2012 for Anhui Province, and 11049 households were included in this study. The study compared differences in out-of-pocket expenditure on health care between regions (urban vs. rural areas) and years (2011 vs. 2012) using two-sample t-test, and also investigated the degree of inequality using Lorenz and concentration curves.

**Result:**

Approximately 5% and 8% of total household consumption expenditure was spent on health care for urban and rural populations, respectively. In 2012, the wealthiest 20% of urban and rural population contributed 49.7% and 55.8% of urban and rural total health expenditure respectively, while the poorest 20% took only 4.7% and 4.4%. The concentration curve for out-of-pocket expenditure in 2012 fell below the corresponding concentration curve for 2011 for both urban and rural areas, and the difference between curves for rural areas was greater than that for urban areas.

**Conclusion:**

A substantial and increasing gap in health care expenditures existed between urban and rural areas in Anhui. The health care financing inequality merits ample attention, with need for policymaking to focus on improving the accessibility to essential health care services, particularly for rural and poor residents. This study may provide useful information on low income areas of China.

## Background

The World Health Organization reported that out-of-pocket (OOP) represents the main financing source and contributes significant portions of household spending in low-middle counties [1]. China is no exception. According to the international study conducted in 2002/03, 53.6% (US$133.4), 56.0% (US$81.8), 58.5% (US$134.4) and 56.3% (US$80.2) of total health expenditure (THE) was financed by OOP in China, Malaysia, Philippines and Vietnam, respectively [2]. In addition, the percentage in Mexico from 2001 to 2006 was approximately 50% [3] and 62% in 2006 in Egypt [4]. China's economic reforms, dating from 1978, have resulted in continued income growth and a parallel boom in consumption. Subsequently, China has witnessed a sharp increase in health care expenditure over the past three decades alongside growth in ability to pay and technological advances. One study found that THE per capita in China increased from US$51(computed at the current exchange rate) in 2000 to US$305 in 2011. This translates to an annual increase as high as 17.4%. Additionally, OOP health care expenditure increased from US$21 in 2000 to US$110 in 2011[5]. In 2013, per capita OOP was estimated to be as high as US$376 [6]. In Anhui, annual per capita expenditure on total household consumption increased from US$331 in 2001 to US$1557 in 2011, an approximately fivefold increase [7]. Growth in annual OOP spending has been even more rapid; OOP in 2011 (US$231) was over 10 times that in 2001 (US$23) [7,8]. These figures all point to a continuous trend of rapid growth in the health care expenditure of individual households.

Given China’s urban-rural dualistic economic structure, there are also indications that inequalities between urban and rural areas have been increasing alongside the rapid economic growth [9]. Rural-urban gaps in health care system development have pushed more and better health care resources into urban areas, with urban–rural differences particularly apparent in the health field. A number of reports have documented these disparities in health care use and health outcomes [10,11,12]. Liu *et al*. reported that a widening gap in health status between urban and rural residents was correlated with an increasing difference in income and health care utilization from 1985 to 1993 [11]. A study conducted in 2003 revealed adverse effects on the health status of populations from growing inequality in income distribution, with almost 50% of rural patients who needed inpatient care failing to receive services due to inability to pay [13]. Another study, conducted in 2004 on the status, impact, and reasons for disparity in health resource allocation between rural and urban areas, found that rural residents made up 80% of China’s population, but only used 20% of total health resources; and concluded that this disparity was persistent and growing [14].

The disparity of OOP between the poor and rich has been observed in many counties. In Vietnam 2002, 7.3% of total household expenditures in the poorest 20% were spent on health services, and the percentage in the richest 20% was only 2.7% [15]. The proportion contributed to income of the poorest 20% households was 13.8% and 49.3% in Egypt and Lebanon, respectively, while in the richest 20% it was 4.7% and 5.6%, respectively [4]. In addition, in Thailand the proration of OOP share of household income in underprivileged family was as much as 5–6%, while in other groups it was 1–2% [16]. A study in China discovered that in urban Gansu in 2007, the wealthiest 20% of the population contributed 43.3% of all OOP and the poorest quintile contributed only 5.9%; the respective percentages for rural areas were 59.1% and 1.1% [17]. Based on a unified design between the city and the countryside, urban–rural inequality in health care expenditure in China has attracted considerable attention in recent years. However, little research has been undertaken on inequality at regional level. Only one study has recently investigated differences in medical expenditure between regions, in which the authors found that income inequality and actual provincial government budget deficits were useful in explaining the discrepancy in health expenditure between urban and rural areas [18].

**Fig 1:**
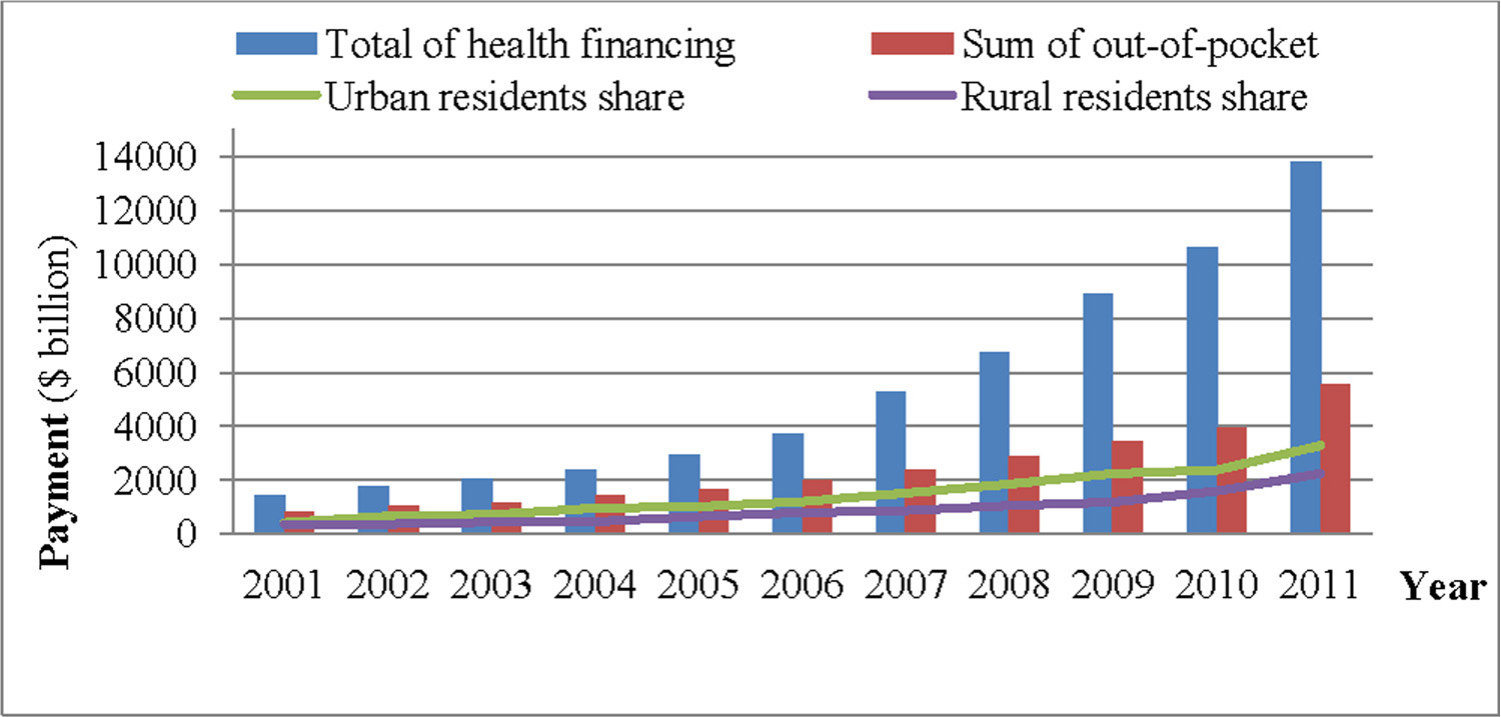
The trend of constitute to out-of-pocket, Anhui (2001–2011).

Anhui Province is located inland in the eastern midlands of China, with a population of 69 million in 2012 [19]. Its rank among the 31 national provinces, in terms of gross domestic product (GDP) per capita, varied between 25^th^ and 26^th^ from 1978 to 2012. Between 2000 and 2011, household consumption expenditure in Anhui increased by 13.9% annually in the urban population and 21.8% in the rural population. Total health financing per capita increased from US$8 in 2001 to US$55 in 2011, with an annual increase rate in OOP of 23% for all areas combined, and 21% and 24% for urban and rural residents respectively (Fig 1). Although the OOP proportion of THE has dropped slightly since 2004, it still accounts for almost 40%. OOP as a percentage spent was consistently more for urban residents than that for rural residents. Since November 2009, the new round of medical and health care reform was launched in China. To improve the public and basic health services, the comprehensive reform of basic health care system was initiated in January 2010 in Anhui. The study aims to examine the OOP diversity in urban-rural and poor-rich between 2011 and 2012 in Anhui, to assist policy-makers in formulating policies and allocating healthcare resources fairly and justly [20].

## Methods

### Data

The majority of data was sourced from the Urban Household Survey and Rural Household Survey of the National Bureau of Statistics of China Survey Office, Anhui Province. These two surveys are conducted yearly on a door-to-door basis by the National Bureau of Statistics. The sampling households in the urban survey are families that live in urban cities and counties, while the sampling of the rural survey consists of families of rural villages. To avoid boredom and ageing and improve the representative, the sampling household will be changed every three years in urban areas and every five years in rural areas. Every family selected for a household survey accurately records the daily incomes and expenditures for all family members, audited on a monthly and quarterly basis. The survey data includes basic household indicators such as the household characteristic, housing, income, living expenditure, and consumption of major consumer goods.

For Urban Household Survey, the stratifying method is used to choose sample districts in cities and towns, and the PPS (probability proportion size) systematic sampling method is used to select sample communities/resident’s committees. Finally the same method is used to select dwellings. Same as in the urban areas, a combination of various sampling approaches is used to identify the survey households for the Rural Household Survey, including PPS systematic sampling method and the systematic random sampling method [21,22]. The 2011/2012 surveys covered 2500 and 3100 households in 15 cities and 31 counties respectively. Information on income and consumption expenditure for an entire year was extracted, including household payments for food, clothing, housing, education, transportation, entertainment, and OOP. OOP included expenditure on consultations, medicines, examinations, treatments, and medical instruments. OOP did not include reimbursement from health insurance, highly specialized health care services, or luxury products, such as ginseng. Consumption expenditure and OOP information was extracted, with personal identity information removed prior to analysis.

The study also obtained figures for total health financing from the Departments of Health in Anhui Province, for the years 2001 and 2011 for the urban and rural areas. The study design and implementation was approved by the Anhui Medical University Research Ethics Committee.

### Indicators and Statistical Analysis

We used number- and curve-based indicators. Number-based indicators were the per-capita total consumption expenditure (TCE), per-capita OOP for health care, OOP as a proportion of TCE, and OOP as a proportion of non-food consumption expenditure (NFCE). The former two indicators were also used to derive two curve-based indicators, a Lorenz curve (LC) and a concentration curve (CC). Consumption expenditure was used as a means to evaluate the household’s economic level, for its more stable, reliable, and easier to obtain than income [23]. Per-capita OOP was used to estimate the absolute health care burden on households; and OOP as a percentage of TCE and NFCE of household was used as an indicator for relative health care burden. Two-sample t-test was used to compare differences in these four indicators between urban and rural areas, and between 2011 and 2012.

Considering the household size and age of the family, per-capita TCE and per-capita OOP were defined as annual consumption expenditure and health care expenditure per adult equivalent (AE) in a household, and the number of AE was defined as follows:

$$\text{AE}={\left(\text{A}+0.5\text{K}\right)}^{0.75}$$

Here, A is the number of adults in the household and K is the number of children (0–14 years old). Households were divided into five quantities according to per adult equivalent consumption expenditure (AECE). Quintile 1 was the poorest 20% of the population, and quintile 5 was the wealthiest 20%.

The two curve-based indicators, LC and CC, have been widely applied in representing wealth and asset distribution [24,25] and together provide a useful representation of absolute and relative inequalities. The cumulative percentage of population is plotted on the x-axis, while the cumulative percentage of consumption payment, on the y-axis, deriving LC to represent wealth or asset distribution. Similarly, CC for OOP is often used to analyze equity, displayed by cumulative health care expenditure share against the cumulative percentage of households in the population (the households are ranked according to their economic level, LC). If p% populations have exactly p% health payment, CC would be represented by a 45° line, known as the line of absolute equality. If the CC lies under the 45° line, distribution is absolute regressive, and if the CC lies under the LC, then the distribution is referred to as relative regressive. As is well known, the greater the distance of the CC from the LC, the greater the inequality [26].

## Results

### Sociodemographic Features of Sampled Households

Table 1 presents characteristics of the sampled population. A total of 11 409 households with 37 605 residents were surveyed. In urban areas, 2391 and 2468 households participated in the 2011 and 2012 surveys, respectively; while in rural areas, 3 095 households participated in the two years. Household size was larger in rural than in urban areas (*p*<0.05) but the difference between two years was not statistically significant. The average ratio of male to female in urban households was 1.12 and 1.13 in 2011 and 2012, respectively. Of the sampled residents, more than 25% of urban residents had completed college education, compared with less than 2% in rural areas (*p*<0.05). The percentage of people over 60 years of age were higher in urban than in rural areas (14.0% vs. 12.9% in 2011, 14.6% vs.13.3% in 2012), and the proportion of rural households was higher in 2012 than in 2011. In contrast, the proportion of people younger than 14 years of age declined from 2011 (19.6%) to 2012 (18.6%) and was lower (13.2% and 13.1% for 2011 and 2012, respectively) in urban areas. Finally, the percentage of elderly among the 5 quintiles had significantly different in both areas in the two years, with the exclusion of urban areas in 2011 (*p* = 0.21).

**Table 1:** Socio-demographic features of sampled populations.

Variables	Description	N (%)			
		Urban 2011 (A)	Rural 2011 (C)	Urban 2012 (B)	Rural 2012 (D)
Household					
	Household (N)	2391 (6724)	3095 (12010)	2468 (6916)	3095 (11955)
Size of household					
	Number per household	2.81	3.88	2.80	3.86
	t (*p*)	^I^ 32.95 (0.00) *	^II^ 0.91 (0.36)	^III^ 0.42 (0.68)	^IV^32.34 (0.00) *
Ratio of sex					
	Male/ female	1.12	n.a.	1.13	n.a.
	t (*p*)	n.a.	n.a.	^III^ –0.26 (0.79)	n.a.
Persons with high education					
	Higher and college school	23.9	1.3	25.0	1.3
	Z (*p*)	^I^ –50.66 (0.00) *	^II^ –0.07 (0.94)	^III^ –1.59 (0.11)	^IV^–52.29 (0.00) *
Age (years)					
	—14	13.2	19.6	13.1	18.6
	15–60	71.7	70.7	71.7	68.1
	61+	14.0	12.9	14.6	13.3
	Z (*p*)	^I^ –10.45 (0.00) *	^II^ –3.63 (0.00) *	^III^ –0.89 (0.37)	^IV^–8.26 (0.00) *
Household quintiles					
	Poorest (Q_1_)	568 (23.8)	619 (20.0)	403 (16.3)	619 (20.0)
	2^nd^	488 (20.4)	619 (20.0)	484 (19.6)	619 (20.0)
	3^rd^	485 (20.3)	619 (20.0)	487 (19.7)	619 (20.0)
	4^th^	454 (19.0)	619 (20.0)	518 (21.0)	619 (20.0)
	Wealthiest (Q_5_)	396 (16.6)	619 (20.0)	576 (23.3)	619 (20.0)
Percentage of elderly in household					
	1^st^ (Q_1_)	14.0	18.2	16.0	17.0
	2^nd^	15.0	14.4	15.0	15.0
	3^rd^	14.0	10.1	15.0	14.0
	4^th^	16.0	10.5	15.0	10.0
	5^th^ (Q_5_)	13.0	9.3	13.0	8.0
	χ^2^(*p*)	14.08 (0.01) *	129.27 (0.00) *	5.92 (0.21)	105.81 (0.00) *

Note: n.a, not applicable

I, A/C

II, C/D

III, A/B

IV, B/D.

### Expenditures by Different Populations

The TCE, OOP and OOP proportion of THE in the 5 quintiles are shown in Table 2. As shown in Table 2, urban households had higher OOP, both in terms of absolute money and proportion of TCE than rural households. OOP as a percentage of TCE was 5.4% (2011) and 5.3% (2012) for urban residents, and 8.6% (2011) and 8.7% (2012) for rural residents. OOP as a percentage of NFCE was 9.6% (2011) and 9.4% (2012) for urban residents, compared with 15.1% and 15.3% for rural residents. In summary, the proportion of OOP of both TCE and NFCE was lower in urban than rural areas. The difference between urban and rural TCE and OOP increased from 2011 (urban–rural TCE difference: US$1522, urban-rural OOP difference: US$52) to 2012 (US$1715, US$62). Similar differences were also found for OOP proportion of TCE and NFCE (TCE from 2011–2012: 3.2% to 3.5%; NFCE from 2011–2012: 5.5% to 5.9%).

**Table 2:** Mean value of household consumption and health expenditures by socio-economic status.

Variable	Quintile	2011		2012	
		Urban (A)	Rural (C)	Urban (B)	Rural (D)
Per-capita TCE ($)					
	1^st^ (Q_1_)	1005	240	1045	268
	2^nd^	1547	407	1575	444
	3^rd^	2018	567	2057	627
	4^th^	2641	790	2715	896
	5^th^ (Q_5_)	4742	1641	5002	2022
	Average	2251	729	2623	908
	Difference ^a^	1522		1715	
	Ratio ^b^	3.1		2.9	
	t (*p*)	^I^ 48.63 (0.00) *	^II^ –5.41 (0.00) *	^III^ –6.36 (0.00) *	^IV^47.96 (0.00) *
Per-capita (OOP) ($)					
	1^st^ (Q_1_)	48	17	46	19
	2^nd^	75	32	78	37
	3^rd^	107	47	104	53
	4^th^	185	70	152	83
	5^th^ (Q_5_)	221	176	314	242
	Average	120	68	149	87
	Difference ^a^	52		62	
	Ratio ^b^	1.8		1.7	
	t (*p*)	^I^ 4.14 (0.00) *	^II^ –2.43 (0.02)*	^III^ –2.338 (0.02) *	^IV^3.52 (0.00) *
OOP share of TCE (%)					
	1^st^ (Q_1_)	4.8	7.0	4.4	6.8
	2^nd^	4.8	7.8	5.1	8.3
	3^rd^	5.3	8.3	5.0	8.4
	4^th^	7.0	8.9	5.6	9.2
	5^th^ (Q_5_)	5.0	10.9	6.1	11.1
	Average	5.4	8.6	5.2	8.7
	Difference ^a^	-3.2		-3.5	
	Ratio ^b^	0.6		0.6	
	t (*p*)	^I^ –10.47 (0.00) *	^II^ 22.62 (0.00) *	^III^ –0.18 (0.86)	^IV^12.82 (0.00) *
OOP share of NFCE (%)					
	1^st^ (Q_1_)	9.6	15.9	8.9	15.0
	2^nd^	9.3	15.2	9.8	15.9
	3^rd^	9.6	14.8	9.7	15.1
	4^th^	11.9	14.5	9.6	15.0
	5^th^ (Q_5_)	7.7	15.2	8.9	15.4
	Average	9.6	15.1	9.4	15.3
	Difference ^a^	-5.5		-5.9	
	Ratio ^b^	0.6		0.6	
	t (*p*)	^I^ –11.57 (0.00) *	^II^ 26.71 (0.00) *	^III^ 0.55 (0.58)	^IV^15.25 (0.00) *

Note

a, urban–rural

b, urban/rural

I, A/C

II, C/D

III, A/B

IV, B/D.

Table 3 presents the contribution of the households in 5 quintiles to TCE, OOP and OOP proportion of THE. As shown in Table 3, there were significant differences in TCE and OOP between different areas in 2011 and 2012 (*p*<0.05). OOP as a proportion of TCE and NFCE exhibited significant differences between all the subgroups except for urban households between 2011 and 2012 (*p* = 0.86 and 0.58, respectively). TCE and OOP were higher in wealthier than poorer quintiles, with the highest expenditure on consumption and healthcare observed in quintile 5. However, the highest OOP as a proportion of TCE and NFCE was found in quintiles 4 and 5. TCE by the wealthiest quintile was 3–7 times that of the poorest quintile, especially for rural residents in 2012, with a proportion of 47.5% and 6.0% in 2011 and 2012 respectively. By comparison, difference in OOP between quintiles was greater than difference in TCE. The ratio (the wealthiest quintile to the poorest quintile) in TCE and OOP in urban areas in 2011 was 3.3 and 3.6 respectively; and in rural areas in 2012, 7.5 and 12.8. A large difference in proportion share in OOP between the wealthiest quintile and the poorest quintile was observed, with 51.4% of OOP spending for rural areas in 2012; 55.8% of OOP was spent by the wealthiest quintile and higher than its population percentage (17.0%), while the poorest quintile contributed to only 4.4% (with the population percentage of 23.7%). In both areas and two years, the proportions of Q4 and Q5 contributed to OOP were higher than their population proportion, and the proportion of Q1, 2 and 3 were opposite. Furthermore, the largest OOP proportions of TCE were observed in quintiles 4 and 5, in both areas for both years. OOP as a proportion of NFCE showed similar trends.

**Table 3:** Distribution of expenditures by socio-economic status (%).

Variable	Quintile	2011		2012	
		Urban	Rural	Urban	Rural
Per-capita TCE					
	1^st^ (Q_1_)	10.6	6.6	6.5	6.3
	2^nd^	14.0	11.2	11.8	10.4
	3^rd^	18.2	15.6	15.5	14.7
	4^th^	22.3	21.7	21.7	21.0
	5^th^ (Q_5_)	34.9	45.0	44.5	47.5
	Difference ^a^	24.3	38.4	38.0	41.2
	Ratio ^b^	3.3	6.8	6.8	7.5
Per-capita OOP					
	1^st^ (Q_1_)	8.8	5.1	4.7	4.4
	2^nd^	12.2	9.3	10.3	8.5
	3^rd^	17.8	13.8	13.6	12.2
	4^th^	29.5	20.5	21.8	19.2
	5^th^ (Q_5_)	31.7	51.3	49.7	55.8
	Difference ^a^	22.9	46.2	45.0	51.5
	Ratio ^b^	3.6	10.1	10.7	12.8

Note

a = Q5-Q1

b = Q5/Q1.

Comparing 2012 with 2011, the ratio (the wealthiest quintile to the poorest quintile) in OOP increased from 3.6 to 10.7 for urban areas, and 10.1 to 12.8 for rural areas. OOP as a proportion of TCE and NFCE among the wealthier groups was even higher than that for poorer groups. In rural areas, the wealthiest quintile accounted for more than half (55.8%) of THE in 2012. As a whole, expenditure distribution across different quintiles was more even in urban than rural areas and more inequitable in 2012 than 2011.

### Concentration Curve of Expenditure

**Fig 2:**
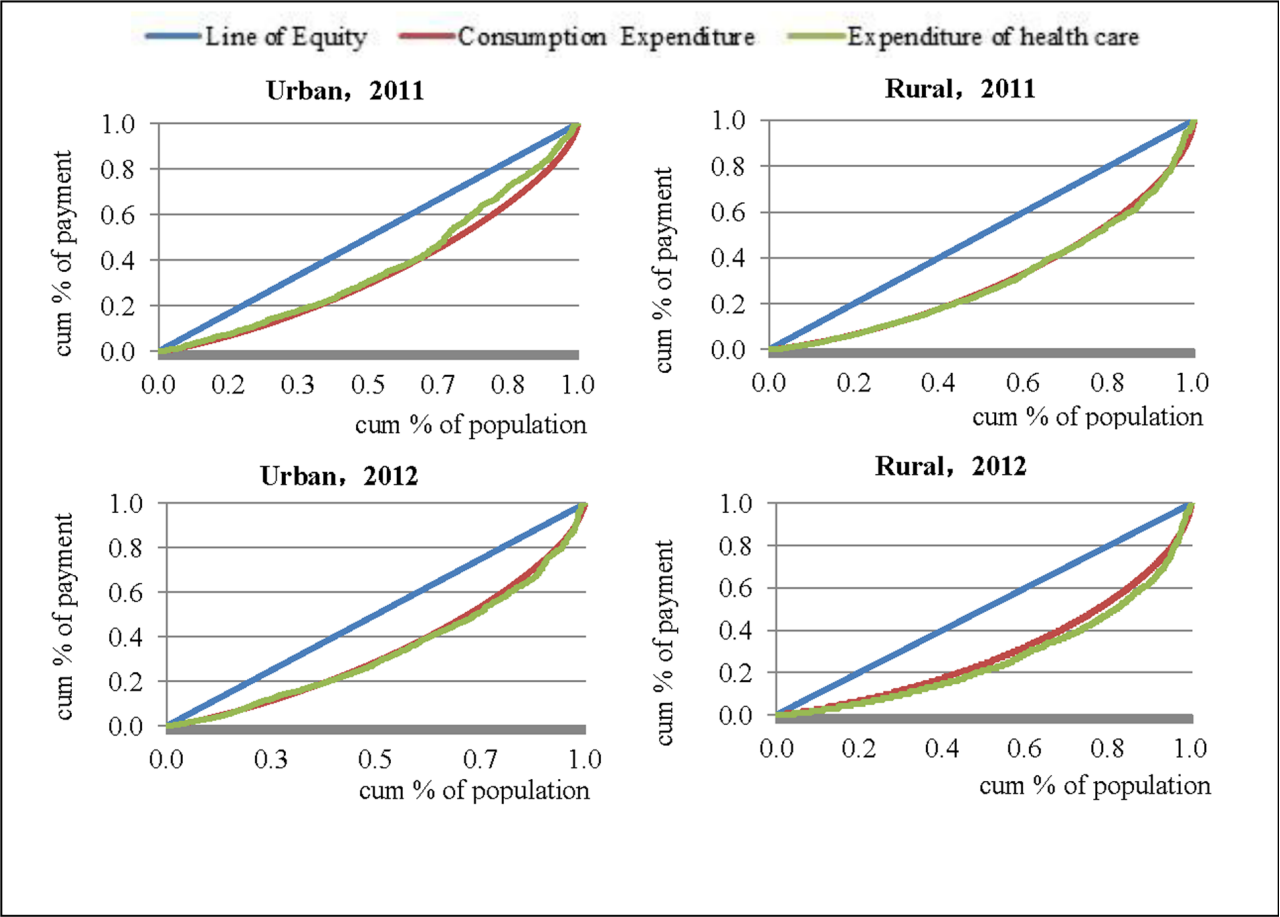
The Lorenz cure and concentration curve of health care expenditure.

Fig 2 illustrates the CC for health care (OOP) and the LC (TCE). The CCs for OOP expenditure fell below the equity line, almost coinciding with the LC line for the two years and areas. It should be noted that the final section of the CC for urban residents fell from above to under the LC for 2011, and the CC for urban households was closer to the equity line for 2012, compared with that for rural households, indicating that the gaps between LC and CC for OOP were greater for urban areas than for rural areas. The distances between CCs were larger in 2012 than in 2011. It implied that the equality of OOP for rural areas was worse than that for urban areas, and the inequality in 2012 was greater than that in 2011.

## Discussion

In terms of relative health care expenditure, our study revealed a couple of noteworthy trends between 2001 and 2011. First, although TCE increased 1.9-fold in total (or by 13.9% annually) over the past 10 years among urban populations, and 2.5-fold (or by 21.8% annually) among rural populations, health care expenditure increased much more sharply during the same period, 3.4-fold and 29.6-fold respectively. As a result, health care expenditure as a proportion of TCE witnessed substantial growth in both urban (from 4.4% to 6.9%) and rural (from 4.9% to 8.9%) areas [27]. So it appears the study population had not benefited from China’s decade-long economic boost; on the contrary, it had suffered substantially. Second, compared with urban areas, rural areas were hit much harder during the study period. In 2001, health care expenditure as a proportion of TCE was higher in urban than rural areas (urban: 4.4%, rural: 4.9%; while in 2011, the gap between the same share in urban and rural areas became larger (urban: 6.0%, rural: 8.9%).

Our findings regarding OOP are consistent with the above observations. Although OOP as a percentage of THE fell from 2001 to 2011 by 40.1%, it was higher than the Chinese average (34.8%). Urban residents contributed 59.4% of total OOP, which is higher than the proportion of the population living in urban areas, (44.8%); while 55.2% of rural residents took on 40.6% of total OOP. As pointed out by a World Health Organization report in 2005, health is extremely inequitable when more than 50% of THE is OOP, and equity is partially achieved for selective services only when this proportion falls to between 30% and 50% [19]. It is evident, therefore, that health care financing in Anhui needs further improvement, to provide financial protection to households, particularly in rural areas. Our study found that the OOP of urban residents was greater in absolute money but lower as a proportion of health care expenditure, compared with that of rural residents. This indicates a significant socioeconomic disparity. That may be explained by the fact that urban people can afford higher prices and higher levels of health care services because of their greater ability-to-pay, while for rural residents even small health care expenditures can be a catastrophic shock to their household economics [2]. The LCs and CCs for OOP provide additional information on this disparity: OOP was more unevenly distributed in urban than rural areas; and the gap between the LCs and CCs has been increased in 2012 compared to 2011, the regressive nature of which was more pronounced in rural than urban areas.

The study also found substantial discrepancies between groups of differing economic status. OOP by the wealthiest 20% population in urban and rural areas was 10.6 and 12.7 times, respectively, that of the poorest 20%. OOP of TCE and NFCE proportion was lower in urban areas. Similar results were found in previous research in Taiwan [28] and India [29], and more recently in western China [30]. With higher ability and willness-to-pay for health care, urban residents are likely to use higher level, more expensive and a greater amount of health care services; while, when faced with income limitations, some residents reduce their utilization of health care services. A previous study showed that elderly spent more on health care, especially over the age of 65 years [31]; however, this is not evidenced in this study. As the study showed, the percentage of elderly was higher in the poorer quintile with lower OOP, which might be explained to some degree by the above fact. Other individuals with income limitations decide to sacrifice their consumption expenditure in other categories, such as education, investment or production, to meet immediate health needs [30]. It has been previously shown that an increase of 100 Chinese Yuan in medical expenditure leads to a 0.22% reduction in farming investment in rural China [32]; therefore, high health expenditure damages the economic and physical wellbeing of households. Then they may even forego long-term benefits by using savings or going into debt.

Since implementation of the comprehensive reform on basic health care system in 2009, Anhui issued a series of new policies on the reform, including the pattern of payment, zero-profit on drug sales, equal access to basic public health services, first contact care of the community, and so on. From 2009–2010, the reform’s focus was on laying the foundation; however, the focus has shifted to boosting the quality of services during the 2011–2015 periods. The financial burden of health care on households (especially the poor and rural ones) continues to increase at the early stage of the reform and must be viewed as a priority issue for government policy. Enhancing the gate-keeping function of primary care services is an important measure for strengthening financial protection for poor and rural households, and the community’s first option should be generally and continuously promoted

### Limitation

One limitation of this paper is that it only demonstrates the current situation of OOP health care payment after the comprehensive health reforms, but there is limited evidence to connect the change of health care expenditure directly to the reforms due to lack of comparison to the OOP health care before 2009.

## Conclusion

Our study revealed prevalent health expenditure burden and inequities in Anhui, China. OOP as a proportion of THE has increased rapidly between 2001 and 2011. Annual per capita OOP expenditure was 6% and 8% of TCE in urban and rural areas in 2011 and 2012, respectively, and wealthier groups sustained proportionally higher OOP than poorer quintiles. Inequality was more apparent in rural than urban areas. Health care financing inequality merits ample attention, with a need for policymaking to focus on enhancing the proportion of government and social financing in THE. We also suggest reducing health care costs by improving accessibility to basic health services and implementing virtually the policy of community as first option, particularly for rural and poor residents.

## Abbreviations

THE: Total health expenditure
OOP: out-of-pocket health care expenditure
TCE: total household consumption expenditure
NFCE: non-food consumption expenditure
AECE: adult equivalent consumption expenditure
